# Marker assisted selection for *Varroa destructor* resistance in New Zealand honey bees

**DOI:** 10.1371/journal.pone.0273289

**Published:** 2022-09-16

**Authors:** James Sainsbury, Tomi E. Nemeth, Maria Baldo, Mateusz Jochym, Crystal Felman, Mark Goodwin, Michael Lumsden, David Pattemore, Ferenc Jeanplong

**Affiliations:** 1 The New Zealand Institute for Plant & Food Research Limited, Hamilton, New Zealand; 2 Coast to Coast Bees Limited, Te Kowhai, Hamilton, New Zealand; University of Leipzig Faculty of Life Sciences: Universitat Leipzig Fakultat fur Lebenswissenschaften, GERMANY

## Abstract

*Varroa destructor* is a honey bee (*Apis mellifera*) parasite identified as one of the leading causes of overwintering colony loss in New Zealand. It has been shown that a naturally occurring heritable trait, “*Varroa* Sensitive Hygiene” (VSH), confers an advantage to colonies by increasing behaviours that limit the survival and reproduction of *Varroa* mites. The SNP 9–9224292 is an adenine/guanine (A/G) polymorphism on chromosome 9 of *Apis mellifera* where the G allele was observed to be associated with VSH behaviour in North American honey bees. In this study, we sought to determine if selection for the G allele of SNP 9–9224292 could decrease *Varroa* mite infestation of New Zealand honey bee (*Apis mellifera ligustica*) colonies. We genotyped queens and tracked their colonies over summer before measuring *Varroa* levels at the point of autumn *Varroa* treatment. The mean *Varroa* population level in colonies headed by queens that carry two copies of VSH associated G allele of SNP 9–9224292 was 28.5% (P<0.05) lower compared with colonies headed by queens with two copies of non-VSH associated A alleles. Although a significant reduction in mite infestation was achieved in treatment colonies, conventional *Varroa* treatment was still required for adequate *Varroa* control. Considering the open mating of queens used and a lack of drift control in this study, this VSH SNP shows promise for marker assisted selection of New Zealand honey bees when aiming for innate *Varroa* control traits.

## Introduction

*Varroa destructor* is a honey bee (*Apis mellifera*) ectoparasite identified as one of the leading causes of overwintering colony loss in New Zealand [1]. Beekeepers use different methods or combination of methods to control *Varroa* infestation such as drone brood removal, brood interruption, heat treatment, organic or synthetic chemicals. However, the efficacy, safety, costs and labour associated with these methods are quite variable. Commercial beekeeping operations in New Zealand typically rely on synthetic miticides such as amitraz, fluvalinate or flumethrin to control *Varroa* mites, although persistent and widespread instances of mite resistance to synthetic miticides have been reported worldwide [2–4]. Issues with emerging mite resistance and residues of these miticides in honey bee products [5,6], and increasing customer preference for organic honey [7] stimulated ongoing research to explore sustainable strategies including biological control [8], gene drive technology [9] and the selection of *Varroa* resistant honey bee lines in controlling mite infestation [10].

*Varroa* mite resistance of honey bees can manifest at the colony and individual level. Swarming and absconding provide a colony level mechanism that creates a broodless period which breaks the cycle of mite reproduction [11,12]. There are individual and social defence mechanisms against ectoparasites such as self- and allo-grooming that can remove phoretic mites, and hygienic behaviour which aims to remove dead or diseased brood [13]. In 2007, Harris introduced a new specific term, the so-called *Varroa* Sensitive Hygiene (VSH) for honey bees that preferentially remove mite infested pupae aged less than five days post-capping [14]. This is a *Varroa*-specific behaviour but some elements of it are thought to be shared with suppressed mite reproduction, a different mechanism that has shown high correlation with VSH behaviour in some populations [15]. The components of VSH such as the identification of parasitized brood, uncapping and removal of infested pupae are believed to be encoded by multiple genes and performed by different house bees of the colony [16]. Hence, VSH is regarded as a heritable trait of *Apis mellifera* similarly to hygienic behaviour that confers an advantage to colonies by increasing behaviours that limit the survival and reproduction of *Varroa* mites [17–19]. Therefore, the VSH trait can be incorporated into queen breeding programmes to offer beekeepers a sustainable strategy for controlling *Varroa* mites [20]. However, measuring bee behaviour in a functioning colony can be a time- and cost-intensive procedure, thus it can be a limiting factor in breeding programs [21].

Marker Assisted Selection (MAS) is a breeding strategy that has been successfully applied in other livestock species to enhance selection processes and has been proposed as a method to improve honey bee stocks [22,23]. The requirement to apply MAS is a genetic marker linked with a respective trait. There is an adenine/guanine (A/G) single nucleotide polymorphism (SNP 9–9224292) located on chromosome 9 at nucleotide position 9224292 of the honey bee genome (assembly Amel 4.0) that has been observed in association with VSH behaviour in North American stocks of *Apis mellifera* [24]. Authors used genotypes of 1,340 SNPs of the *Apis mellifera* genome to construct a high-resolution genetic map and employed interval mapping to interrogate association between genotypes and performance in VSH behaviour. Direct observation of individually labelled nursing bees was used to identify VSH behaviour on mite infested combs involving the perforation of wax capping of mite infested cells, enlargement of holes of perforated capping and removal of pupa from the fully uncapped cells. Candidate genes in this chromosomal location are involved in olfaction, vision, learning and memory [24]. Although the above SNP has the potential to be used in MAS against *Varroa* mites as identified in an experimental bee population, it has not been demonstrated whether it does work at standard field conditions. Furthermore, given the polygenic nature of VSH behaviour it is unclear whether selection for a single genetic marker can reduce the level of *Varroa* mite infestation in New Zealand honey bee colonies.

The aim of this study was to determine if selection of honey bee queens for the VSH associated G allele of SNP 9–9224292 could lead to reduced *Varroa* mite infestation of New Zealand honey bee colonies (*Apis mellifera ligustica*). To achieve this aim, naturally infested hives headed by queens homozygote for either the G or A allele were set up and monitored during summer, and then total *Varroa* infestation levels were determined at the time of autumn mite treatment.

## Materials and methods

### Genotyping of SNP 9–9224292

Mated queens of less than one year of age were sampled at Coast to Coast Bees Ltd (CTOC), a commercial beekeeping operation by taking a wing clipping (about 1/3 of a wing) and storing it in ethanol at -20°C. The HotSHOT protocol was used to extract genomic DNA from the wing clippings [25]. Briefly, following the removal of storage ethanol, 75μl of alkaline lysis reagent (25mM NaOH, 0.2mM disodium EDTA, pH 12 without adjustment) was added to the wing clipping and incubated at 95°C for 30min. Samples were cooled down to 4°C and 75μl of neutralizing reagent (40mM Tris-HCl, pH 5 without adjustment) was added and mixed by gentle pipetting. Two microliters of this preparation were used as a DNA template in 20μl of PCR reaction volume. The queens were genotyped for SNP 9–9224292 using a PCR amplification/restriction digest assay as described by Kirrane et al. [26]. The DNA region of this SNP was amplified using the PCR primers: forward 5’-CGCGTGTATGTGTGTATTTACAAAGTTCGG-3’ and reverse 5’-TACTTGCTCGTCCATCGTCCATA-3’. The PCR product was digested with Hpy188i (NEB, MA, USA) restriction enzyme, separated in a 2.5% agarose gel and stained with ethidium bromide. The G allele was observed as a band of 207 bp and the A allele a band of 178 bp. Queens homozygous for the VSH associated G allele were designated as “treatment queens” while queens homozygous for the non-VSH associated A allele were designated as “control queens”. These experimental, genotyped queens were marked on the thorax and used to re-queen colonies and placed in a single apiary to standardise environmental conditions. The queens at CTOC were bred in-house and obtained from local queen breeders which were recorded to include as a covariate in the analysis. All queens were openly mated, therefore the genotype of drones mated with the genotyped queens were not controlled. It was assumed that the genotype distribution of drones was about 1:1 in the natural environment, therefore the presence of the VSH genotype in workers had become significantly higher in colonies headed by queens homozygous for the G allele (~75% of all alleles is G in workers, 50% GG and 50% GA) compared to colonies headed by queens homozygous for the A allele (~75% of all alleles is A in workers, 50% AA and 50% GA) during the trial. Regardless of the genotype distribution of workers in the recipient colonies, it is plausible to assume that the target genotypes become dominant in “control” and “treatment” colonies during the trial considering the two weeks interval between the time of re-queening and the start of trial, 10-week timeframe of the trial and 4–8 weeks lifespan of worker bees in summer [27]. Testing of heterozygous queens was considered but to keep costs down and hive numbers at an acceptable level for a commercial beekeeping operation they were not included in this trial.

### Colony set up

Two months before the trial started in spring 2017, *Varroa* mite treatment of colonies used in this trial was completed with flumethrin (Bayvarol) according to the manufacturer’s instructions. The efficacy of the spring mite treatment was confirmed in randomly selected colonies using alcohol washes (≤1 mite per 100 bees) at CTOC apiaries. Queens were collected from eight apiaries and genotyped for SNP 9–9224292 as described above. Candidate recipient colonies, previously used for honey production, were assessed to confirm they contained all three stages of brood, had no visible signs of disease and colony strength was equalized to within +/- one frame. Two weeks before the beginning of the trial in the middle of December 2017 (early summer in New Zealand), forty recipient colonies were re-queened with genotyped queens (“treatment” GG queens, n = 18; and “control” AA queens, n = 22) and relocated to a single CTOC registered apiary site in Te Kowhai, New Zealand. Natural *Varroa* mite infestation was allowed to occur in all colonies. Hives consisted of two three-quarter depth boxes placed on a ventilated bottom board (Hive Doctor Smart, Ecrotek, New Zealand) with a queen excluder between the brood box and a honey super with drawn out frames to measure honey production. The weight of honey supers was recorded. Hives were placed in two rows in a north-facing semicircle arrangement. Colonies were inspected fortnightly and fed with the same amount of sugar syrup once at about the middle of the trial when it became apparent that the colonies did not have sufficient honey reserves. No efforts were made to manage inter-colony drift in the research apiary, a phenomenon expected to reduce any genotype effect.

### Determination of *Varroa* levels

At the beginning of January 2018 (summer in New Zealand), when we started the trial, both sides of each frame of every hive were photographed and the colony size (number of bees) was estimated by two observers based on the percentage of the comb surface covered by bees [28]. An alcohol wash provided an estimate of phoretic mites per 100 bees. The total *Varroa* load at the start of the trial was then estimated by multiplying the number of bees in each colony with the phoretic mite rate to provide a covariate (*initial Varroa load*) for subsequent analysis [29]. After about 10 weeks (69 days), the colony size and phoretic mite number were estimated using the same methods mentioned above and the presence of the original queen was confirmed in each colony. Each honey super was weighed as well. Following the hive assessment, flumethrin (Bayvarol) and amitraz (Apivar) strips were applied to each hive according to the manufacturer’s instructions and *Varroa* fall was determined using white pest trays inserted under the ventilated floor board (Hive Doctor Smart^TM^, Ecrotek, New Zealand) smeared with canola oil for four weeks. Pest trays were replaced weekly, and mites were counted manually. The total number of mites were calculated as the sum of the four weekly counts.

### Statistical analysis

For pairwise comparisons, the Welch’s t-test was used and differences at P<0.05 were regarded as statistically significant. To assess the effect of the source of queens, time and genotype of the queens on *Varroa* levels, we employed a Generalized Linear Model with a negative binomial distribution and *log* link function (GLM). The analysis was performed with R ver. 3.5.0 [30] and package MASS [31]. Significance of terms was established with a likelihood ratio test (LRT).

## Results and discussion

The honey bee SNP 9–9224292 was successfully genotyped in 179 mated queens at CTOC using a PCR amplification and restriction endonuclease digest which distinguishes between the G and A alleles [26]. We observed a genotype frequency of 34.1% GG, 22.9% AA and 43% GA in CTOC queen stock. These results suggest that the two alleles are present at about 1:1 ratio in the unselected queen population with a slight positive bias towards the G allele. We speculate that a similar genotype frequency might be found in the unselected workers of the source and recipient colonies as well. The genotype frequency of North American bee stock is yet to be reported.

**Fig 1:**
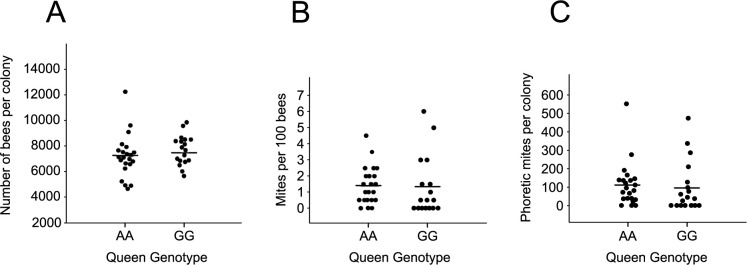
Colony size (A), mite wash (B) and estimated total phoretic mite infestation in colonies (C) headed by treatment (GG) and control (AA) queens at the beginning of the beehive trial. The mean of each data set is represented by a horizontal bar.

At the beginning of the field trial, the colony size and phoretic mite number were estimated in all beehives. The colony size of treatment (GG) and control (AA) colonies was not different (P = 0.28, Fig 1A). There was no difference in the phoretic mite infestation levels between treatment and control colonies using alcohol washes (P = 0.80, Fig 1B). To confirm this observation, we fitted a series of GLMs to the initial *Varroa* load. We found no evidence for differences between the genotype and source of queen, fitted either as simple terms (LRT against null model; genotype: |^2^ = 0.049 at 1 df, p = 0.82; source of queen: |^2^ = 0.049 at 1 df, p = 0.39), or in an interaction (LRT against simplified model: |^2^ = 0.98 at 1 df, p = 0.32). The estimated total number of phoretic mites per colony was not different between treatment and control colonies (P = 0.76, Fig 1C). These data suggest that colony size and natural *Varroa* infestation level were not different between treatment and control colonies at the beginning of the field trial. Also, the results confirmed that *Varroa* mite infestation was at a relatively low level in early summer when we started the experiment.

**Fig 2:**
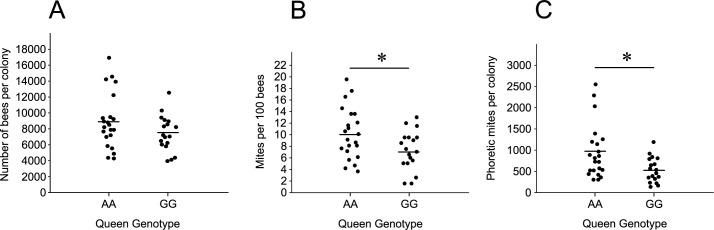
Colony size (A), mite wash (B) and estimated total phoretic mite infestation in colonies (C) headed by treatment (GG) and control (AA) queens at about 10 weeks of the beehive trial. The mean of each data set is represented by a horizontal bar. Asterisks indicate significance between genotypes (*P<0.05).

At about 10 weeks after the beginning of the field trial, the colony size and phoretic mite number were estimated again in all beehives. There was no difference in colony size between treatment and control colonies (P = 0.07, Fig 2A). The number of phoretic mites decreased by 28% in treatment colonies to those of controls (P = 0.028, Fig 2B). The estimated total number of phoretic mites in each colony decreased by 44% in treatment colonies to that of controls (P = 0.011, Fig 2C). Our results indicate that the VSH genotype of the queen resulted in a decrease in the phoretic mite infestation of colonies, but it did not have a significant effect on colony size. It remained unclear if the VSH genotype had an effect on the total number of mites in colonies because an unknown number of reproducing mites was hidden in capped brood cells.

**Fig 3:**
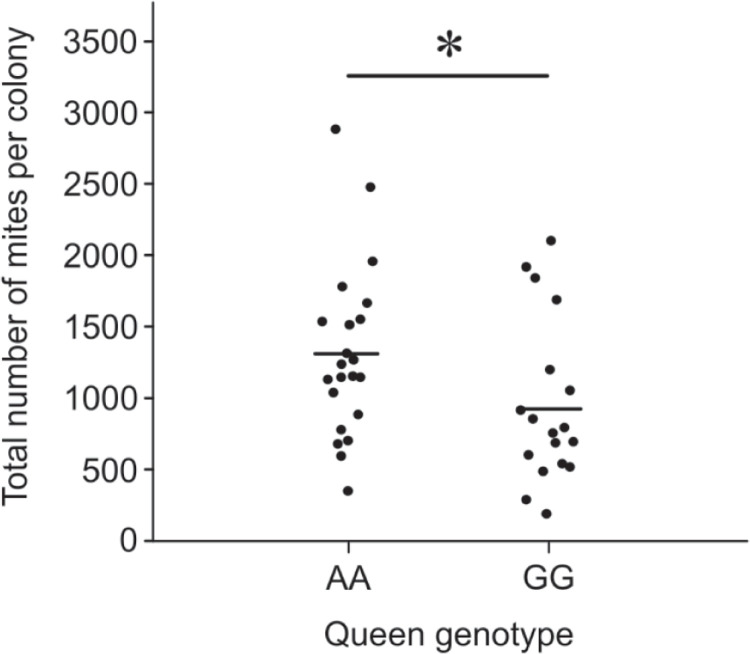
Total mite infestation of colonies headed by treatment (GG) and control (AA) queens at the end of the beehive trial. The mean of each data set is represented by a horizontal bar. Asterisks indicate significance between genotypes (*P<0.05).

To find out the total number of mites (phoretic plus the ones in capped brood cells) in each colony, synthetic miticide strips were placed into all beehives for 4 weeks and the number of *Varroa* mites killed were counted on sticky boards. Subsequently, we fitted a GLM to the estimate of the total *Varroa* counts at the end of the experiment. The model included a three-way interaction between genotype, source of queen, and initial *Varroa* load. In the course of manual model simplification, we established that neither the three-way interaction (LRT: |^2^ = 0.081 at 1 df, p = 0.77) nor any of the two-way interactions (LRT; genotype vs. source of queen: |^2^ = 0.19 at 1 df, p = 0.65; genotype vs initial *Varroa load*: |^2^ = 0.82 at 1 df, p = 0.36; initial *Varroa* load vs. source of queen: |^2^ = 1.05 at 1 df, p = 0.30) were significant. A simple term for the source of the queen was non-significant (LRT: |^2^ = 0.26 at 1 df, p = 0.61), and was thus dropped from the model. We found a significant simple effect of genotype (LRT: |^2^ = 4.92 at 1 df, p = 0.027) showing 28.5% lower *Varroa* infestation level in treatment colonies compared to those of controls (Fig 3). Furthermore, there was a significant simple effect of initial *Varroa* load on the final mite levels (LRT: |^2^ = 10.0 at 1 df, p = 0.0016).

A guanine/adenine polymorphism at the honey bee SNP 9–9224292 has been described in honey bees (*A*. *mellifera*) in North America. From the results it can be seen that this single nucleotide polymorphism also occurs in New Zealand honey bees. In the initial North American study, the guanine allele at SNP 9–9224292 was associated with VSH behaviour, which is a variable trait innate to honey bees that can help control the destructive mite *V*. *destructor*.

We did not measure VSH behaviour directly in this trial which requires extensive colony manipulation and direct observation of bee activity. We used the total level of mite infestation at the time of autumn *Varroa* treatment to assess if the presence of two copies of the VSH associated G alleles of SNP 9–9224292 would provide *Varroa* control in New Zealand honey bees. This method is different from the VSH bioassays others used where mite infested brood was introduced into VSH colonies for either 40 h or one week and the removal of mite infested brood was directly measured [14,21]. Although it is the method of choice in relatively small experiments, they are quite labour intense, and time consuming in large field trials and require multiple mite donor colonies with known high levels of mite infestation. This latter requirement is particularly difficult to manage at a commercial beekeeping operation. Mite washes and mite drops in response to synthetic miticide treatment can only measure phoretic and total mite infestations, respectively. Therefore, we can only speculate that the lower *Varroa* load at the end of the trial might have arisen from stronger VSH behaviour in treatment colonies compared to controls. In support, Ward et al [32] have found significantly lower phoretic mite load in VSH colonies than in control colonies in a three-year long field trial. Furthermore, in a recent study, a positive relationship has been shown between strong VSH behaviour (percentage of mite infested brood removal) and natural mite fall in colonies of Russian honey bees [33]. We predicted that the genotype frequency of G allele associated with VSH behaviour became dominant in treatment colonies by the end of summer which has been shown to coincide with a higher level of VSH activity in mite resistant colonies [34]. These observations suggest that a lower *Varroa* load is associated with a stronger VSH behaviour that may show a seasonal variation as well.

Our results show that the total *Varroa* population was 28.5% lower in the treatment colonies compared with control colonies (Fig 3). This observation is supported with the estimated number of phoretic mites at 10 weeks of the trial which was 28% lower in treatment colonies than those of controls (Fig 2B). The most likely explanation for this difference would be that the treatment colonies might express higher levels of VSH traits significantly suppressing the growth of *Varroa* population. These data are consistent with the VSH behaviour found in North American honey bees carrying G alleles of SNP 9–9224292 [24]. Although we found a strong positive correlation between the autumn *Varroa* load and the VSH genotype of queens, it would require further investigation to confirm an enhanced VSH behaviour of workers in those colonies of New Zealand honey bees. It was also noted that *Varroa* levels were quite variable across colonies regardless of any genotype effect which is not surprising as other factors are known to contribute to *Varroa* population growth such as initial *Varroa* load, visitation of non-natal bees, the number of foragers with mites and viral dynamics in colonies [35–39].

We observed a significant effect of initial *Varroa* level on the final *Varroa* load of colonies which is expected during summer with an expansion of brood development and a subsequent increase in bee population that create ideal conditions for rapid mite reproduction the speed of which is dependent, at least in part, on initial mite infestation. It is well known that it is hard to accurately measure mite infestation when *Varroa* infestation is low using non-destructive methods, particularly in summer when most mites are reproducing in capped cells. Therefore, we cannot entirely rule out the impact of the limited accuracy of initial mite counts on the final *Varroa* load of colonies. However, it is plausible to assume a similar level of horizontal transmission of mites between colonies via drifting of bees in the experimental apiary which is likely to reduce the impact of such variation on initial mite counts. Although we acknowledge these limitations, our statistical analysis showed a significant effect of genotype suggesting a clear difference between the treatment and control colonies related to the genotype of queens.

The experimental apiary located in an area used for grazing of dairy cattle with a limited abundance of nectar source. Monitoring of honey supers during the trial revealed very little honey deposition in supers. This observation was confirmed at the end of the 10-week trial when each honey super was weighed (S1 Table). Even though we were interested in determining if the queen’s genotype had any impact on honey production, it will remain the subject of future work. As regular hive inspections revealed a lack of honey in supers, all colonies were fed with the same amount of sugar syrup once at about the middle of the trial to prevent starvation. Although, we do not know whether it had any impact on the VSH behaviour of colonies of different genotypes, we cannot rule it out.

Interestingly, in a follow up study of Tsuruda et al. [24] in Russian honey bees that are known to be *Varroa* resistant, the opposite A allele of SNP 9–9224292 was associated with VSH behaviour [26]. Authors argued that the trait may not be identical in Russian and North American honey bees and the phenotyping method used in the original QTL study might not be the most appropriate to characterise VSH behaviour [26]. In contrast, our results in New Zealand honey bees have shown a positive correlation between the G alleles of the queen and a reduced *Varroa* load of colonies which is consistent with the findings of Tsuruda et al. [24].

To our knowledge, this is the first study where a positive correlation has been shown between this VSH genetic marker (the G allele of the honey bee SNP 9–9224292) and a reduced *Varroa* load trait at standard colony conditions. All queens used in this trial were openly mated which meant that the genetic contribution of drones to control and treatment colonies was unknown. In agreement with our results, colonies headed by mite-resistant queens that were open-mated with unselected drones showed significantly less *Varroa* mite infestation than colonies headed by control queens [40].

Although our results are encouraging in the fight against *Varroa* mites, the biggest threat against the health and survival of western honey bees, follow-up field trials with a larger cohort of beehives are needed to confirm these findings. These future trials can address some key questions as well that remained unanswered such as genotype frequency of workers and drones, investigation VSH behaviour of workers and honey production. We hypothesize that the *Varroa* infestation of colonies can be further reduced using artificial insemination or controlled mating of virgin queens with drones that carry the VSH associated G allele of SNP 9–9224292. This hypothesis is yet to be tested and potential trade-offs in New Zealand honey bees.

MAS has clear advantages over classical phenotypic selection in honey bee breeding. Rapid genetic improvement can be achieved without running long and expensive field trials, performing complicated and time-consuming bioassays. The VSH marker shows promise to be developed into a cheap molecular diagnostic test for the beekeeping industry. However, more research is required to develop such a MAS programme including the identification of causative genes involved in VSH and associated other SNPs. Simultaneous selection for multiple markers associated with *Varroa* resistance would make MAS more robust. There are other challenges as well that need to be addressed such as education of queen breeders, mating control to maintain high genotype frequency of beneficial markers, availability of cost-effective commercial molecular diagnostic services and prevention of inbreeding in breeding populations.

Although a significant reduction in mite infestation was achieved in treatment colonies, conventional *Varroa* treatment was still required for adequate *Varroa* control. It is plausible to propose that selection for this genetic marker and an increase in the genotype frequency of the VSH marker in the New Zealand honey bee population would still be of value by providing flexibility around timing of conventional treatments and could contribute to an integrated pest management strategy against *Varroa* mites. Therefore, the VSH associated honey bee SNP 9–9224292 shows promise in marker assisted selection of New Zealand honey bees when aiming for innate *Varroa* control traits.

## Supporting information

S1 Table The weight of supers at the end of the 10-week trial. (DOCX)
