# Supplementary material for: Marker assisted selection for Varroa destructor resistance in New Zealand honey bees
Source: PLoS One. 2022 Sep 16;17(9):e0273289. doi: 10.1371/journal.pone.0273289 (PMC9480979; doi:10.1371/journal.pone.0273289)
Supplement: S1 Table — (DOCX) [file pone.0273289.s001.docx]

S1 Table. The weight of supers at the end of the 10-week trial.

| Hive ID number | Genotype | Weight of supers (kg) at Day 1 | Weight of supers (kg) at Day 69 | Change in weight of supers (kg) | Mean | SD | SEM | t-test |
| --- | --- | --- | --- | --- | --- | --- | --- | --- |
| 78 | AA | 7.88 | 7.86 | -0.02 |  |  |  |  |
| 83 | AA | 8.32 | 6.64 | -1.68 |  |  |  |  |
| 107 | AA | 7.3 | 5.8 | -1.5 |  |  |  |  |
| 123 | AA | 7.38 | 6.28 | -1.1 |  |  |  |  |
| 135 | AA | 7.84 | 6.06 | -1.78 |  |  |  |  |
| 142 | AA | 8.24 | 6.52 | -1.72 |  |  |  |  |
| 145 | AA | 8.28 | 6.74 | -1.54 |  |  |  |  |
| 146 | AA | 8.82 | 7.24 | -1.58 |  |  |  |  |
| 147 | AA | 8.12 | 10.5 | 2.38 |  |  |  |  |
| 150 | AA | 8.24 | 6.7 | -1.54 |  |  |  |  |
| 151 | AA | 8 | 6.3 | -1.7 |  |  |  |  |
| 178 | AA | 8.24 | 6.72 | -1.52 |  |  |  |  |
| 180 | AA | 8.42 | 6.58 | -1.84 |  |  |  |  |
| 181 | AA | 7.76 | 6.1 | -1.66 |  |  |  |  |
| 183 | AA | 8.1 | 6.8 | -1.3 |  |  |  |  |
| 184 | AA | 8.1 | 6.42 | -1.68 |  |  |  |  |
| 187 | AA | 8.06 | 6.74 | -1.32 |  |  |  |  |
| 188 | AA | 8.26 | 6.6 | -1.66 |  |  |  |  |
| 189 | AA | 8.86 | 6.72 | -2.14 |  |  |  |  |
| 193 | AA | 7.7 | 6.96 | -0.74 |  |  |  |  |
| 195 | AA | 8.06 | 6.1 | -1.96 |  |  |  |  |
| 197 | AA | 7.9 | 6.52 | -1.38 | -1.32 | 0.94 | 0.19937 |  |
| 50 | GG | 8.54 | 6.42 | -2.12 |  |  |  |  |
| 53 | GG | 8.56 | 7.12 | -1.44 |  |  |  |  |
| 61 | GG | 7.96 | 7.04 | -0.92 |  |  |  |  |
| 71 | GG | 7.92 | 6.44 | -1.48 |  |  |  |  |
| 72 | GG | 7.92 | 6.42 | -1.5 |  |  |  |  |
| 79 | GG | 8.08 | 6.62 | -1.46 |  |  |  |  |
| 99 | GG | 8.06 | 6.3 | -1.76 |  |  |  |  |
| 119 | GG | 8.14 | 6.5 | -1.64 |  |  |  |  |
| 121 | GG | 7.84 | 6.76 | -1.08 |  |  |  |  |
| 122 | GG | 7.72 | 6.2 | -1.52 |  |  |  |  |
| 130 | GG | 8.62 | 6.9 | -1.72 |  |  |  |  |
| 139 | GG | 7.8 | 6.58 | -1.22 |  |  |  |  |
| 140 | GG | 8.7 | 6.82 | -1.88 |  |  |  |  |
| 144 | GG | 8.86 | 7.3 | -1.56 |  |  |  |  |
| 154 | GG | 8.22 | 6.64 | -1.58 |  |  |  |  |
| 175 | GG | 7.78 | 6.12 | -1.66 |  |  |  |  |
| 32 | GG | 7.92 | 6.38 | -1.54 |  |  |  |  |
| 104 | GG | 7.82 | 6.72 | -1.1 | -1.51 | 0.29 | 0.069 | 0.3693 |
